# Study of inter- and intra-individual variations in the salivary microbiota

**DOI:** 10.1186/1471-2164-11-523

**Published:** 2010-09-28

**Authors:** Vladimir Lazarevic, Katrine Whiteson, David Hernandez, Patrice François, Jacques Schrenzel

**Affiliations:** 1 Genomic Research Laboratory, Geneva University Hospitals, Rue Gabrielle-Perret-Gentil 4, CH-1211 Geneva 14, Switzerland

## Abstract

**Background:**

Oral bacterial communities contain species that promote health and others that have been implicated in oral and/or systemic diseases. Culture-independent approaches provide the best means to assess the diversity of oral bacteria because most of them remain uncultivable.

**Results:**

The salivary microbiota from five adults was analyzed at three time-points by means of the 454 pyrosequencing technology. The V1-V3 region of the bacterial 16S rRNA genes was amplified by PCR using saliva lysates and broad-range primers. The bar-coded PCR products were pooled and sequenced unidirectionally to cover the V3 hypervariable region. Of 50,708 obtained sequences, 31,860 passed the quality control. Non-bacterial sequences (2.2%) were removed leaving 31,170 reads. Samples were dominated by seven major phyla: members of Firmicutes, Proteobacteria, Actinobacteria, Bacteroidetes and candidate division TM7 were identified in all samples; Fusobacteria and Spirochaetes were identified in all individuals, but not at all time-points. The dataset was represented by 3,011 distinct sequences (100%-ID phylotypes) of ~215 nucleotides and 583 phylotypes defined at ≥97% identity (97%-ID phylotypes). We compared saliva samples from different individuals in terms of the phylogeny of their microbial communities. Based on the presence and absence of phylotypes defined at 100% or 97% identity thresholds, samples from each subject formed separate clusters. Among individual taxa, phylum Bacteroidetes and order Clostridiales (Firmicutes) were the best indicators of intraindividual similarity of the salivary flora over time. Fifteen out of 81 genera constituted 73 to 94% of the total sequences present in different samples. Of these, 8 were shared by all time points of all individuals, while 15-25 genera were present in all three time-points of different individuals. Representatives of the class Sphingobacteria, order Sphingobacteriales and family Clostridiaceae were found only in one subject.

**Conclusions:**

The salivary microbial community appeared to be stable over at least 5 days, allowing for subject-specific grouping using UniFrac. Inclusion of all available samples from more distant time points (up to 29 days) confirmed this observation. Samples taken at closer time intervals were not necessarily more similar than samples obtained across longer sampling times. These results point to the persistence of subject-specific taxa whose frequency fluctuates between the time points. Genus *Gemella*, identified in all time-points of all individuals, was not defined as a core-microbiome genus in previous studies of salivary bacterial communities. Human oral microbiome studies are still in their infancy and larger-scale projects are required to better define individual and universal oral microbiome core.

## Background

Bacterial communities in the mouth have a significant impact on the general health by either preventing or causing infections. Recent data suggest a causative relationship between oral microbiota profiles and specific diseases including periodontitis [1,2]. Bacterial species that are more prevalent in healthy subjects as well as those that have higher counts in diseased individuals have been identified. Based on an extensive literature review, Siqueira and Rôças [3] concluded that some oral pathologies may have a polymicrobial aetiology and that different types of infection are represented by various mixed bacterial consortia.

Most of the bacteria in saliva are attached to exfoliated human epithelial cells [4]. In addition to its clinical importance as a diagnostic indicator of oral cancer [5] and possibly other diseases, the human salivary microbiome may provide insights into human populations structure and migrations. Surprisingly, initial studies suggest there is little geographic structure of the human salivary microbiome, although specific bacterial genera e.g. *Serratia *and *Enterobacter *vary significantly across geographic locations [6].

Using traditional and molecular approaches, more than 700 bacterial species have been identified in the human oral cavity and approximately half of them are not yet cultivated [7]. The study of complex oral microbiotas with a classical approach would require new culturing technologies whose development is laborious and intrinsically limited. Metagenomics offers an attractive alternative, bypassing the need to culture bacteria. The sequencing of the entire microbiome, used to characterize communities dominated by a small number of species [8] cannot be readily applied for the analysis of highly-complex human microbiota. Therefore, high-throughput sequencing of amplified partial 16S rDNA sequences of a bacterial community currently provides the best compromise between sequence coverage, analytical speed and experimental costs.

Recent studies of oral microbiota using high-throughput sequencing estimated the number of species-level phylotypes between 540 and about 10,000 [9-11]. However, these figures were obtained using different sequencing coverage, sampling different anatomical sites and analyzing samples pooled from different number of individuals. Therefore, not all of the identified taxa are expected to be present in the same subject and at the same time [7]. In the current study, we assessed the inter- and intra-individual variations of salivary microbiota, by means of a culture-independent approach based on the pyrosequencing of the bacterial 16S rDNA V3 region. We compared salivary bacterial communities of five individuals at three time-points using 16S rDNA pyrosequencing in order to assess their short-term stability and interindividual differences.

## Results and Discussion

### Taxonomic analysis of the salivary microbiota

We explored the microbial diversity of the saliva samples from five individuals by targeting the 16S rDNA hypervariable V3 region. Of 50,708 obtained reads, 31,860 passed the quality control. They were submitted to the MG-RAST server [12] for taxonomic analysis. The BLAST-based MG-RAST analysis with a minimum alignment length of 105, the RDP dataset option and a maximum e-value of 10^-30 ^removed 690 sequences (2.2%), leaving 31,170 sequences which were further analyzed. The majority of removed sequences were identical or nearly-identical to human sequences and two sequences were recognized by the MG-RAST as chimeras. One additional sequence was recognized as a putative chimera after multiple sequence alignment (see below).

**Figure 1:**
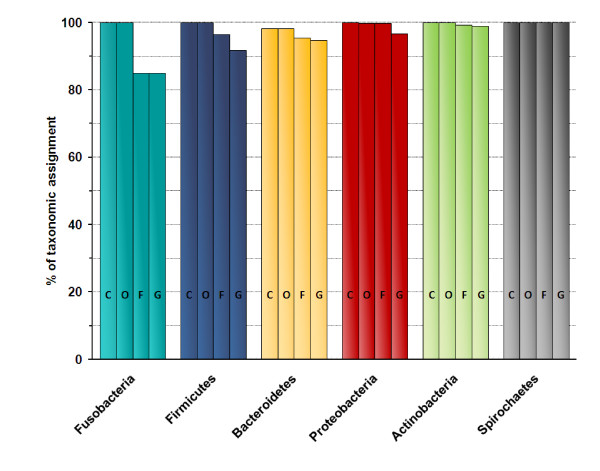
**Proportion of taxonomic assignments under the phylum level**. Bars represent the reads assigned to each of the four taxonomic levels for each major phylum. Their heights represent the percentage of reads that can be placed at a given level of taxonomy using the MG-RAST server. C, class; O, order; F, family; G, genus.

The ability to identify taxa from class down to the genus level varied between phyla but was relatively high. The proportion of sequences that could be confidently placed at the genus level using MG-RAST was 85% for Fusobacteria and over 90% for the five other major phyla (Figure 1). For comparison, 16S rDNA amplicons were analyzed using the RDP classifier with a 80% confidence level [13]. In the RDP-based taxonomic analysis, 64% of sequences assigned to Protobacteria were placed at the genus level. For the five other major phyla the taxonomic assignments at the genus level reached over 90% (not presented).

### Estimation of pyrosequencing errors

To get an estimate of the pyrosequencing errors we calculated the potential errors that can be derived from the most abundant sequence in the entire dataset. This 216-nt long sequence, which matches the relevant 16S rDNA segment of several species of genus *Streptococcus *exactly, occurred 3291 times and was found in all samples. Then we identified sequences that: (i) differed from the most frequent sequence by deleting, inserting or changing any single nucleotide and (ii) lacked exact matches in the reference database. As expected, nucleotide substitutions were the rarest error type with 8 examples (6 distinct sequences). Deletions were more frequent with 26 counts (19 distinct sequences), followed by insertions represented by 58 sequences (35 distinct sequences), which is similar to the trend observed by Huse et al. [14]. The longest homopolymer stretch associated with a putative insertion or deletion was a 4T which became 5T (3 occurrences). All potential error products represented together 2.8% of the most abundant sequence. However, this may be an overestimation since the single-read accuracy of pyrosequencing with the GS FLX System is 99.5% and the majority of errors are undercalling or overcalling the length of homopolymeric stretches [15].

The impact of pyrosequencing errors on classification has been shown to be very small: an insertion/deletion rate of 2% would affect classification of 0.2% reads [16]. In line with this observation, all 92 single-nucleotide derivatives of the most occurring sequence were (correctly) classified using RDP classifier as genus *Streptococcus *with over 98% confidence.

### Composition and variation of the salivary bacterial community

**Figure 2:**
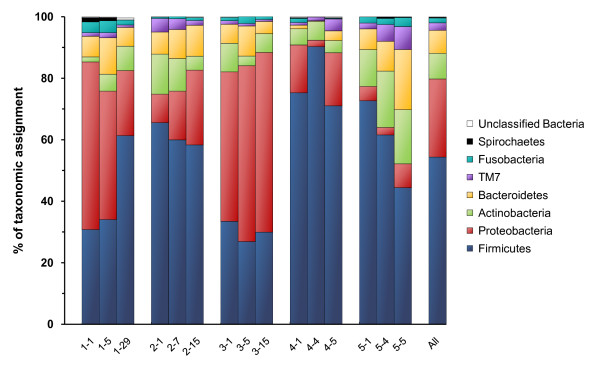
**Relative abundance of predominant phyla across 15 microbiomes from 5 subjects**. Bacterial phyla are indicated by the colour mode. Rare "cyanobacteria" identified in samples 1-5, 1-29 and 5-1 are not depicted. The rightmost column designated as "All" corresponds to the average of phyla frequency in individual samples. Sample numbers include subject ID, hyphen and the follow up time (days) after the first sampling time point (day 1).

Samples are dominated by seven major phyla (Figure 2). Members of Firmicutes, Proteobacteria, Actinobacteria, Bacteroidetes and candidate division TM7 were identified in all samples. Fusobacteria and Spirochaetes, which had the lowest average frequency, were not found in some samples possibly because they were present under the detection limit of the assay. These 7 phyla were also abundant in other oral samples assessed by means of phyloarrays, sequencing of the 16S rDNA clones and 16S rDNA amplicon pyrosequencing [6,9-11,17,18]. The phyla abundances were 99.5-100% identical between MG-RAST (Figure 2) and RDP Classifier (not presented). "Cyanobacterial" sequences found in two individuals corresponded to plant chloroplast sequences. They were most likely transient "contaminants" of the mouth linked to food intake or exposure to airborne pollen [19]. Forty-two sequences recognized as bacterial were not placed into any phylum.

The most abundant phylum in each sample was either Firmicutes or Proteobacteria (Figure 2). In four subjects there was a clear dominance of one of these over the other phylum in the three time-point samples. Subject #4 had the lowest Bacteroidetes content. In subject #5, a low proportion of Proteobacteria was associated with a higher abundance of Actinobacteria.

**Figure 3:**
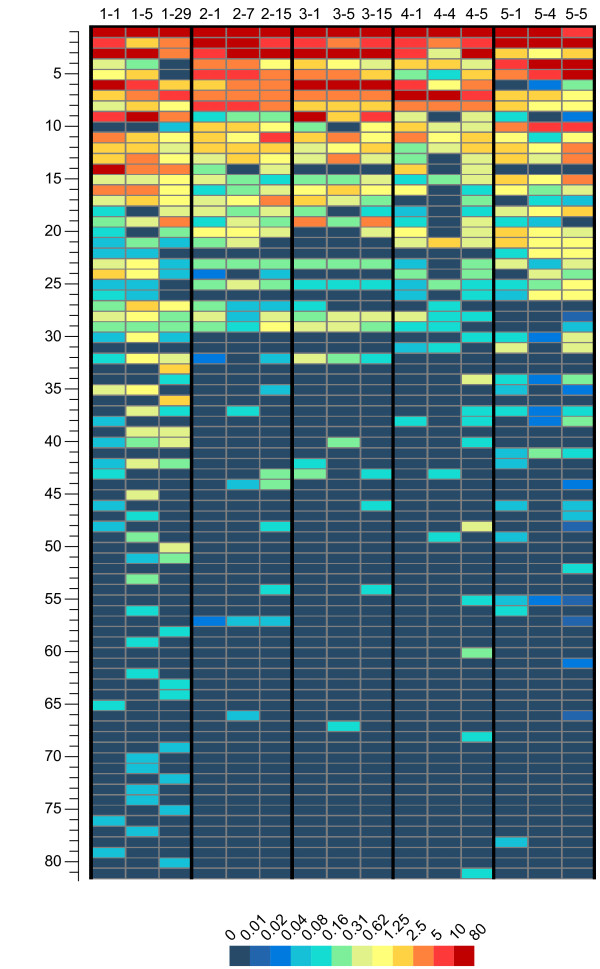
**Relative abundance of bacterial genera across samples**. Rows 1 to 81 correspond to genera listed in Additional file 1, Supplementary Table 1. Each column represents one sample. The abundance (%) is indicated according to the scale at the bottom of the plot. The sequences assigned to genera cover 85-97% of total sequences in individual samples.

A total of 81 genera were identified by the MG-RAST server (Additional File 1). Among them 19 were neither reported in previous studies of oral microbiotas assessed by high-throughput sequencing [6,9,10,17] nor listed in the Human Oral Microbiome Database (HOMD; http://www.homd.org). For individual samples, 3 to 15% of sequences could not be assigned to any of the genera. These sequences, representing 9.5% of the full dataset, were placed into a total of 35 taxa above the genus level. Although specific genera varied significantly in frequencies among the same and across different individuals, many had a consistent presence across most samples (Figure 3). Fifteen out of 81 genera constituted 73 to 94% of the total sequences present in different samples. Eight genera (*Streptococcus*, *Veillonella*, *Haemophilu*s, *Granulicatella*, *Actinomyces*, *Gemella*, *Campylobacter*, *Selenomonas) *were found in the three time points of all subjects. Of these eight genera, all except *Gemella *were found previously in microbiomes of all of the three investigated subjects assessed by a pyrosequencing approach [11]. Between 15 and 25 genera were present in all three time-points of different individuals. Fifty-six genera were relatively rare, occurring at a frequency lower than 1.25% across all samples.

The dataset was represented by 3012 phylotypes defined at 100% identity (100%-ID phylotypes or distinct sequences). One 100%-ID phylotype was discarded because it was placed more distantly than the standard archaeal *Methanocaldococcus jannaschii *DSM 2661 outgroup sequence in the MUSCLE alignment-based clustering. The BLAST and RDP analyses showed that this sequence was obviously chimeric, consisting of two distinct domains. The 5' domain (residues 1-175) was assigned to Veillonellaceae (100% RDP confidence) whereas the remaining 3' domain (residues 176-219) corresponded to Lachnospiraceae (69% RDP confidence).

The number of genera in each subject ranged from 23 to 46 and the number of OTUs defined at 97% identity (97%-ID phylotypes or OTUs_003_) ranged from 56 to 259 for the different samples. However, due to different sampling sizes, these figures cannot be readily compared (Additional File 2).

**Figure 4:**
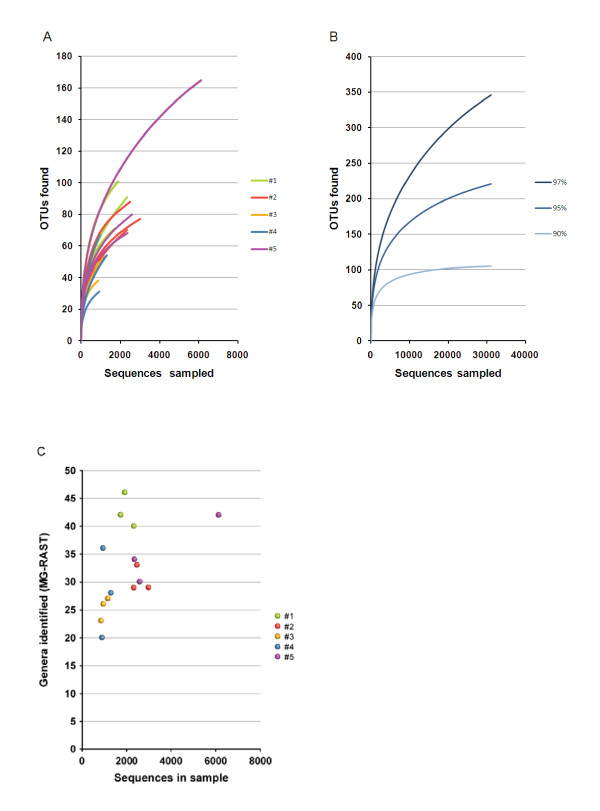
**Number of phylotypes and genera as function of the total number of sequences**. **A**. Rarefaction curves of individual samples. Curves were generated at the 97%-ID cutoff using RDP pyrosequencing pipeline [24]. The three samples from the same subject are represented by the same colour. **B**. Rarefaction curves of the pooled dataset. OTUs with ≥97%, ≥95% and ≥90% pairwise sequence identity generated using RDP pyrosequencing pipeline [24] are arbitrarily assumed to form the same species, genus and family respectively. **C. **Number of genera. Taxonomic composition was identified using MG-RAST. The three samples from the same subject are represented by symbols of the same colour.

A steep slope on the rarefaction curve (Figures 4A and 4B) suggests that a large fraction of the species richness has not been sampled. The number of 97%-ID phylotypes appears to be dependent on the total number of sequences in a given sample. This trend has been lost on the genus-level and higher-taxonomic levels. As shown in Figure 4C, the genus-richness was higher in subject #1 than in subject #2, although the samples from the latter showed better coverage. Of course, the number of phylotypes may be overestimated due to PCR errors, undetected PCR chimeras and sequencing errors [20]. On the other hand, inefficient lysis of some bacteria and the presence of sequences deviating from the broad-range 16S rDNA primers targets will lead to underestimation of bacterial diversity. Chao1 richness estimations (Additional File 2) were the lowest for samples with lowest coverage. This underestimation of less sampled communities has been observed in other studies [21].

**Figure 5:**
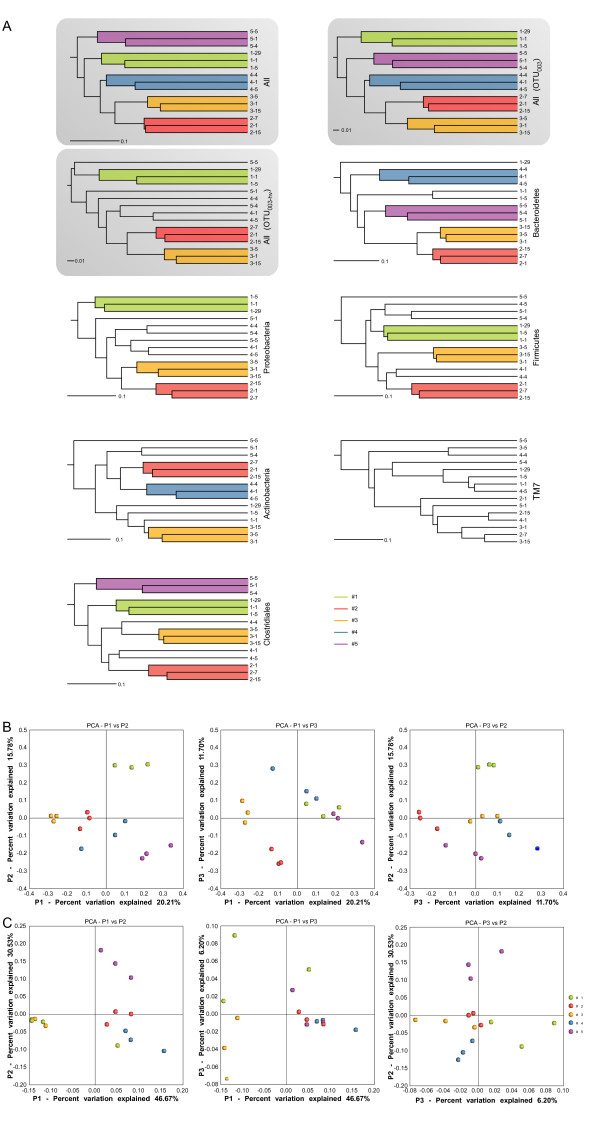
**Comparison of the salivary microbiotas**. **A**. Hierarchical clustering trees were generated using unweighted UniFrac based on the presence or absence of all 3011 phylotypes (All) defined at 100% identity or subsets including indicated phylum or order Clostridiales. The trees based on 583 phylotypes defined at 97% identity and their derivatives obtained by the removal of hypervariable regions are designated All OTU_003_, and All OTU_003-hv_, respectively. Clusters formed by the three time points of the same subject are colour-shaded. PCoA analysis based on unweighted (**B**) or weighted (including abundance) UniFrac and 100%-ID phylotypes (**C**). Samples from the same subject are represented by the same colour.

We compared saliva samples from different individuals in terms of the phylogeny of their microbial communities using UniFrac, where larger values are assigned to changes in more distant taxa [22]. For this purpose, phylotypes including 16S rDNA hypervariable positions were defined at 100% identity, i.e. a threshold which is higher than the widely assumed species-level of 97-99% [23]. We used this stringent cutoff in order to calculate distances between samples with the highest possible resolution. Based on the presence and absence of 100%-ID phylotypes (unweighted UniFrac), samples from each subject formed distinct clusters (Figure 5A). Hierarchical clustering of UniFrac distances based on phylotypes defined at an identity threshold of 97% resulted in grouping of samples for 5 subjects when the alignment of OTUs_003 _was performed with MUSCLE and included hypervariable positions (Figure 5A). However, when hypervariable 16S rDNA positions were removed from the analysis of OTUs_003_, resulting in OTUs_003-hv _with 187 comparable positions, samples of only 3 subjects were grouped. This removal of hypervariable regions is conducted automatically as part of the alignment procedure of the RDP alignment algorithm [24], which allows for tidy comparison of sequences with the same number of positions, but also may eliminate valuable information. Distances between samples were also subject to principal coordinates analysis (PCoA) based on the presence/absence of 100%-ID phylotypes (unweighted UniFrac; Figure 5B) or including their abundance (weighted UniFrac; Figure 5C). The results of the PCoA show that samples from the same subject formed clusters with generally little overlap and that samples from different subjects were better separated using unweighted UniFrac.

We also investigated whether the samples could be grouped based on sequences of individual phyla. We constructed phylogenetic trees from the sequences for each of the 5 phyla identified across all samples (Firmicutes, Proteobacteria, Bacteroidetes, Actinobacteria and TM7). Individual samples were then clustered within each of the five trees using the unweighted UniFrac method. The results presented in Figure 5A show that intrapersonal differences were globally smaller than interpersonal ones for all examined phyla except TM7. Of all phyla, Bacteroidetes were the best indicator of intraindividual similarity over time; the three time points of four subjects and two of one subject grouped closely. When bacterial communities were compared based on sequences of each of the six individual orders present in all samples, the best clustering by subject was found for Clostridiales (Figure 5A).

Different clustering patterns were obtained for different taxon-specific sequences. Samples from subjects #2 and #3 formed distinct clusters in 4/5 phylum-specific datasets. Samples from subject #2 were also grouped in 6/6 analyzed order subsets (not presented) which suggests the high stability of the microbiota in this individual. Taken together, these results indicate that the rate of oral microbiota changes differs between taxonomic groups of bacteria as well as between individuals.

### Common and subject-specific taxa

Analysis of the gastro-intestinal microbiota revealed the existence of a "universal core" consisting of species present in all (investigated) individuals and "individual core" representing the stable colonizers in a single subject [25]. To explore this concept, we calculated the frequency of all taxa and searched for those that were detected in all time points of all individuals and those that were shared between all time points of some (1-4) individuals but absent in all time-points of other individuals (subject-specific taxa). The proportion of taxa belonging to the universal core decreased when moving from the higher taxonomic level (phylum) down to 100%-ID phylotypes, whereas the frequency of subject-specific taxa were more stable across the different taxonomic levels (Table 1). The universal core was represented by 0.3% of distinct sequences and 1.9% of OTUs_003_, which corresponds to 23.3% and 37.6% of the full dataset, respectively. The number of subject-specific phylotypes, calculated at 100% identity, was 4-fold higher than at the 97% identity. Therefore, the interindividual diversity includes the presence of subject-specific phylotypes which are not detected using a 97%-ID phylotype identity cutoff. For instance, 11 100%-ID phylotypes belonging to genus *Veillonella *were shared in all time points of 1 to 3 subjects, whereas no 97%-ID phylotypes of this genus were found to be subject-specific. However, we cannot exclude that some low-abundance subject-specific 100%-ID phylotypes identified were due to sequence errors. In view of the possibility that different subject may be preferentially colonized by different strains of the same species, studies looking to understand the variation in human oral microbiotas may benefit from an identity cutoff greater than 97% in the formation of OTUs. Studies of microbial diversity in the ocean have also used phylotypes that are defined more stringently, with 100% identity, and found that rare organisms are more useful for clustering members from similar communities than more abundant phyla, although they found that this was true both for the 100%-ID phylotypes and a less stringent ~94%-phylotype [26].

**Table 1:** Number of common and subject-specific taxa

	Total # of taxa	# of common taxa (%)	# of subject-specific taxa (%)
Phylum	8	5 (62.5)	0 (0)
Class	15	4 (26.7)	1 (6.7)
Order	32	6 (18.8)	1 (3.1)
Family	57	9 (15.8)	2 (3.5)
Genus	81	8 (9.9)	1 (1.2)
OTU_003_	583	11 (1.9)	17 (2.9)
Distinct sequences (100%-ID phylotypes)	3011	9 (0.3)	69 (2.3)

Taxa found in the three time-point samples of all subjects are designated as common. Subject-specific taxa correspond to those found in the three time-point samples from some subjects and absent in the three time-point samples from the others. Taxa (phylum to genus) were identified using MG-RAST with a minimum alignment length of 105, the RDP dataset option and a maximum e-value of 10^-30 ^[12]. OTUs_003 _were defined using CD-HIT [33], and include the hypervariable regions.

Firmicutes, which are generally the most abundant in the oral metagenome, also have the highest numbers of both universal core and subject-specific phylotype representatives (Additional File 3). Representatives of the class Sphingobacteria, order Sphingobacteriales and family Clostridiaceae were found only in subject #1. In the three time points from subject #1, Sphingobacteria had a frequency of 0.04%, 0.1% and 3.7%. Therefore, Sphingobacteria may be temporal colonizers of susceptible individuals. Sequences corresponding to family Peptostreptococcaceae were detected in all samples except those from subject #3. The only subject-specific genus was *Olsenella*. This genus, found only in subject 5, is diverse, encompassing five different OTUs_003_.

## Conclusions

The salivary bacterial community comparisons using UniFrac revealed that samples from the same individual were clustered, i.e. the salivary microbial community appeared to be stable over at least 5 days. Including samples from more distant time points (15-29 days) performed for three subjects confirmed subject-specific grouping. Moreover, our results show that within the same subject, samples taken at closer time intervals were not necessarily more similar than samples obtained across longer sampling times. These results point to the persistence of subject-specific taxa whose frequency fluctuates between the time points. Because of its relative stability, salivary microbiota may be potentially applied as an alternative or complementary approach in forensics for person identification, as it has been recently proposed for skin bacterial communities [27].

Recently, Costello et al. [19] showed that whole-body bacterial communities may be perfectly clustered by host. The three-month time point samples share many taxa, and the oral microbiota are less variable than other investigated body sites. Indeed, in another study twenty-six percent of distinct sequences were shared in oral microbiomes when single samples of three unrelated individuals were compared [11].

Although the present study did not reach a stable value for phylotype richness, several universal core taxa were identified and putative subject-specific taxa were proposed. A deeper sample coverage is expected to increase the number of universal core taxa whereas the effect on subject-specific taxa frequency remains more difficult to predict: A richer sampling depth may reveal new subject-specific taxa members, and some of those defined in this study may no longer appear specific to a given individual or group of individuals. The fact that the same genera are not uniformly considered as universal core members across different studies shows that the metagenomic studies of oral microbiota require larger-scale high-throughput approaches to better define their individual and universal core.

Although the stringent phylotype identity level cutoff of 100% inflates diversity estimates due to pyrosequencing errors [28] it may, as shown in this study, lead to a better clustering of samples by subject than a 97%-ID phylotype-based approach which includes the removal of hypervariable 16S rDNA regions. Applying the latter procedure (partly in order to minimize the influence of sequencing errors) some of the sample diversity is masked. Therefore, the impact of the sequence alignment procedure and the identity threshold used for phylotype grouping on clustering of bacterial communities may depend not only on the frequency of sequencing errors but also on the bacterial community composition.

Factors influencing the oral microbiota composition include age, gender, dietary habits, smoking, oral hygiene, use of antibiotics and, probably, genetic factors. Since salivary microbiota analysis revealed subject-specific grouping over time, it will greatly benefit the field to conduct a long-term survey of a large number of subjects in order to provide insight into the impact of different factors and the dynamics of the microbiota changes. Improvements in high-throughput sequencing techniques, including longer and more accurate reads, will enable better classification of bacteria. Direct metagenome sequencing without a PCR amplification step will eventually provide a less biased measure of the microbial diversity.

## Methods

### Sampling

Unstimulated saliva was obtained from five adult individuals with informed consent. Individuals without obvious signs of oral disease recruited between the laboratory staff were as follows: subject 1, 44-year, male, non-smoker; subject 2, 30-year, pregnant female with well-controlled Type 1 diabetes, non-smoker; subject 3, 34-year, male, non-smoker; subject 4, 30-year, male, smoker; subject 5, male, 30-year, smoker. Samples were taken between 10 and 11 am at three time-points ranging within a 29-day period. Samples were collected by spitting in sterile plastic 50-ml tubes, 100 μL saliva was mixed with the same volume of 2× lysis buffer [Tris 20 mM, EDTA 2 mM (pH 8), Tween 1%] and kept frozen at -20°C until processing. We added proteinase K (Fermentas) 200 μg/ml and incubated samples for 2.5 hours at 55°C [1]. Proteinase K was inactivated by a 10 min incubation at 95°C and the samples were stored at -20°C. The lysis procedure used allows for the detection of many hard-to-lyse species [29].

### PCR primers and conditions

We aligned 753 16S rDNA sequences from the HOMD (October 2008) using MAFFT (-FFT-NS-2, v6.531b) [30]. Primers were selected from the conserved areas of the alignment flanking the V1-V3 region in order to match most sequences. With a 100% match, primers 8Fhomd (5'-GAGTTTGATCMTGGCTCAG) and 534RhomdDEGa (5'-CCGCGRCTGCTGGCAC) produced 723 and 741 hits, respectively, or 713 (94.7%) of the HOMD sequences. Species coverage was over 90% for the phyla Proteobacteria, Firmicutes, Fusobacteria, Bacteroidetes, Spirochaetes and TM7, and 84.7% for the phylum Actinobacteria. The PCR primers were not designed to amplify single HOMD representatives of phyla Chlamydiae and SR1. In a more general way, the 16S forward and reverse primers used produced 81.3% and 96.23% hits in the RDP database [13]. The V1-3 amplicons corresponded to *E. coli *16S rDNA positions 28 to 514, excluding primer sequence.

PCR amplification was carried out in two steps. The first PCR included 2 μL of lysate and 0.5 μM of each forward (8Fhomd) and reverse (534RhomdDEGa) primer in 25 μl PrimeStar HS Premix (Takara). The samples were run for 11 cycles using the following parameters: 98°C for 10 s, 60°C for 15 s, and 72°C for 1 min.

The second PCR contained 0.4-4 μl aliquot from the first reaction and 0.5 μM of both forward B-8fhomd (5'-gccttgccagcccgctcag-*ac*-GAGTTTGATCMTGGCTCAG-3') and reverse A-[601-to-615]-534RhomdDEGa primer (gcctccctcgcgccatcag-NNNNNNNN*-at-*CCGCGRCTGCTGGCAC-3') in 50 μl of PrimeStar HS Premix. The composite PCR primers included: (i) the 454 Life Science 19-base adaptors A (lowercase, underlined) or B (lowercase); (ii) an eight-base sample specific barcode sequence (NNNNNNNN; designated 601 to 615 in [31]); (iii) the sequence of the broad range 16S forward or reverse primer (uppercase) used in the first PCR, and (iv) a dinucleotide sequence (lowercase italic) introduced between the 16S primer and the barcode sequence designed to prevent pairing of different barcodes with rDNA targets. The amplicons were generated in PCR reactions using 28 cycles of 98°C for 10 seconds, 60°C for 15 seconds, and 72°C for 1 min. The negative control was amplified by 35 PCR cycles. PCR products were purified using the MinElute PCR purification kit (Qiagen). Subsequently, 1 μl of the amplified reaction mix was run on the Agilent 2100 Bioanalyzer using a DNA1000 lab chip to confirm the proper size of the generated products and assess their concentration. Hundred ng of each of the purified sample were pooled and sequenced on a Genome Sequencer FLX system (Roche).

### Informatic analysis

Sequences containing uncalled bases, incorrect primer sequences or runs of ≥10 identical nucleotides were removed. Reads with the 16S rDNA reverse oligonucleotide sequence CCGCGRCTGCTGGCGC, containing G instead of A at the penultimate position of the 3' end, were relatively frequent (35%). They are likely due to a sequencing artefact and were not removed from the dataset if other quality criteria were met. After trimming primer sequences, reads shorter than 200 nt were discarded.

After removing sequence duplicates, we created a multiple alignment of the sequences using MUSCLE [32] (using the following parameters: -maxiters 2 and -diags). Sequences corresponding to *E. coli *16S rDNA positions 300-514 were extracted from the multiple alignment and each distinct sequence was assigned as a 100%-ID phylotype. Sequences were assigned to representative phylotypes at 97% identity (OTUs_003_) using CD-HIT [33] with a minimum coverage of 99%. Distances between 100%-ID and 97%-ID phylotypes were calculated using MUSCLE (-maxiters 2 and -diags) [32]. Alternatively, the 97%-ID phylotypes were aligned using Aligner of the RDP pyrosequencing pipeline [24] and the hypervariable regions were removed leaving 187 comparable positions. Then, a phylogenetic tree of these phylotypes (OTUs_003-hv_) was constructed using FastTree [34]. Bacterial diversity was assessed using RDP pyrosequencing tools: Aligner, Complete Linkage Clustering, Rarefaction, Shannon Index and Chao1 Estimator [24,35]. Clustering and principal coordinates analysis were carried out using UniFrac [22].

Trimmed dataset (31,169 reads) is publicly available at the MG-RAST repository [12] under ID 4445506.3. The fasta identifiers of this dataset are described in Additional file 4. Data will also be available through the GenBank Short Read Archive via accession number SRA012505.

## Authors' contributions

KW and VL produced the 16S rDNA sequences. VL and DH carried out the bioinformatic analysis. VL, KW, PF and JS contributed to the experimental design and drafting of the manuscript. All authors approved the final manuscript.

## Supplementary Material

Additional file 1 **Relative abundance of genera**. Click here for file

Additional file 2 **Diversity estimates for the bacteria in salivary samples based on V3 amplicon sequences**. Click here for file

Additional file 3 **Taxonomic positions of universal core and subject-specific phylotypes defined at 100% and 97% identity**. Click here for file

Additional file 4 **Description of the fasta identifiers in the trimmed dataset**. Click here for file
