# Supplementary material for: Study of inter- and intra-individual variations in the salivary microbiota
Source: BMC Genomics. 2010 Sep 28;11:523. doi: 10.1186/1471-2164-11-523 (PMC2997015; doi:10.1186/1471-2164-11-523)
Supplement: Additional file 1 — Relative abundance of genera. [file 1471-2164-11-523-S1.DOC]

**Add 1**

| # | **Genus** | **Avg Frequency (%)** |
| --- | --- | --- |
| 1 | *Streptococcus* | 25.7506 |
| 2 | *Veillonella* | 11.7109 |
| 3 | *Haemophilus* | 8.8048 |
| 4 | *Atopobium* | 6.2741 |
| 5 | *Prevotella* | 5.8891 |
| 6 | *Neisseria* | 4.4008 |
| 7 | *Granulicatella* | 3.3070 |
| 8 | *Actinomyces* | 2.7874 |
| 9 | *Lautropia* | 2.7136 |
| 10 | *Megasphaera* | 2.5757 |
| 11 | *Gemella* | 2.0978 |
| 12 | *Campylobacter* | 2.0464 |
| 13 | *Porphyromonas* | 1.8989 |
| 14 | *Actinobacillus* | 1.4178 |
| 15 | *Selenomonas* | 1.1066 |
| 16 | *Fusobacterium* | 0.8821 |
| 17 | *Capnocytophaga* | 0.7185 |
| 18 | *Cetobacterium ** | 0.7025 |
| 19 | *Rothia* | 0.6383 |
| 20 | *Roseburia ** | 0.5581 |
| 21 | *Erysipelothrix* | 0.5100 |
| 22 | *Anaeroglobus* | 0.3111 |
| 23 | *Tannerella* | 0.2951 |
| 24 | *Treponema* | 0.2823 |
| 25 | *Eubacterium* | 0.2791 |
| 26 | *Schwartzia* | 0.2662 |
| 27 | *Propionibacterium* | 0.2534 |
| 28 | *Bergeyella* | 0.2309 |
| 29 | *Kingella* | 0.2245 |
| 30 | *Filifactor* | 0.1347 |
| 31 | *Peptostreptococcus* | 0.1219 |
| 32 | *Corynebacterium* | 0.1091 |
| 33 | *Planomicrobium ** | 0.1059 |
| 34 | *Catonella* | 0.0898 |
| 35 | *Dysgonomonas ** | 0.0802 |
| 36 | *Pedobacter ** | 0.0802 |
| 37 | *Mogibacterium* | 0.0770 |
| 38 | *Shuttleworthia* | 0.0609 |
| 39 | *Kocuria* | 0.0577 |
| 40 | *Acinetobacter* | 0.0545 |
| 41 | *Olsenella* | 0.0481 |
| 42 | *Staphylococcus* | 0.0481 |
| 43 | *Cardiobacterium* | 0.0385 |
| 44 | *Centipeda* | 0.0321 |
| 45 | *Lactococcus* | 0.0321 |
| 46 | *Actinobaculum* | 0.0225 |
| 47 | *Aminobacterium ** | 0.0225 |
| 48 | *Johnsonella* | 0.0225 |
| 49 | *Moraxella* | 0.0225 |
| 50 | *Porphyrobacter ** | 0.0225 |
| 51 | *Methylobacterium ** | 0.0160 |
| 52 | *Rhodococcus* | 0.0160 |
| 53 | *Brevundimonas ** | 0.0128 |
| 54 | *Chryseobacterium* | 0.0128 |
| 55 | *Cryptobacterium* | 0.0128 |
| 56 | *Mycoplasma* | 0.0128 |
| 57 | *Peptoniphilus* | 0.0128 |
| 58 | *Acetobacter ** | 0.0064 |
| 59 | *Acidovorax* | 0.0064 |
| 60 | *Brochothrix* | 0.0064 |
| 61 | *Catellatospora ** | 0.0064 |
| 62 | *Dialister* | 0.0064 |
| 63 | *Microbacterium* | 0.0064 |
| 64 | *Paracoccus* | 0.0064 |
| 65 | *Pseudomonas* | 0.0064 |
| 66 | *Tetrasphaera* | 0.0064 |
| 67 | *Acholeplasma* | 0.0032 |
| 68 | *Allisonella ** | 0.0032 |
| 69 | *Brachybacterium ** | 0.0032 |
| 70 | *Desulfobulbus* | 0.0032 |
| 71 | *Exiguobacterium* | 0.0032 |
| 72 | *Flavobacterium ** | 0.0032 |
| 73 | *Friedmanniella ** | 0.0032 |
| 74 | *Lactobacillus* | 0.0032 |
| 75 | *Leucobacter ** | 0.0032 |
| 76 | *Massilia ** | 0.0032 |
| 77 | *Oceanimonas ** | 0.0032 |
| 78 | *Pantoea* | 0.0032 |
| 79 | *Shewanella* | 0.0032 |
| 80 | *Sphingomonas* | 0.0032 |
| 81 | *Tetragenococcus* | 0.0032 |

Average genera frequency for 15 samples are represented. Taxa that have not been listed in previous large-scale bacterial oral community studies [6, 9-11, 17] and in the HOMD are marked by an asterisk. Sequences classified as “T7 genera inceratae sedis” were considered as unidentified at the genus level.
