# Supplementary material for: Study of inter- and intra-individual variations in the salivary microbiota
Source: BMC Genomics. 2010 Sep 28;11:523. doi: 10.1186/1471-2164-11-523 (PMC2997015; doi:10.1186/1471-2164-11-523)
Supplement: Additional file 2 — Diversity estimates for the bacteria in salivary samples based on V3 amplicon sequences. [file 1471-2164-11-523-S2.DOC]

|  |  | Number of identified taxa | | | | | | | Statistical characteristics at ≥97% identity^a^ | | | | | | | |
| --- | --- | --- | --- | --- | --- | --- | --- | --- | --- | --- | --- | --- | --- | --- | --- | --- |
| Sample ID | Number of sequences | Phylum | Class | Order | Family | Genus | Distinct sequences | Number of clusters at 97%- dientity including hypervariable positions (OTU_003_)^b^ | Number of sequences^c^ | Number of clusters (OTU_003-hv)_ | Chao | LCI95 | UCI95 | H' | varH | E |
| 1-1 | 2331 | 7 | 13 | 19 | 32 | 40 | 376 | 142 | 2327 | 91 | 181 | 127 | 317 | 2.9 | 0.00083 | 0.652 |
| 1-5 | 1926 | 8 | 15 | 25 | 39 | 46 | 324 | 149 | 1909 | 101 | 130 | 112 | 175 | 3.4 | 0.00095 | 0.736 |
| 1-29 | 1753 | 8 | 14 | 20 | 34 | 42 | 261 | 108 | 1752 | 71 | 85 | 75 | 114 | 2.8 | 0.00139 | 0.660 |
| 2-1 | 2999 | 7 | 11 | 14 | 24 | 29 | 430 | 141 | 2995 | 77 | 119 | 92 | 199 | 2.7 | 0.00070 | 0.624 |
| 2-7 | 2339 | 6 | 10 | 13 | 22 | 29 | 332 | 128 | 2337 | 70 | 108 | 84 | 173 | 2.8 | 0.00074 | 0.661 |
| 2-15 | 2487 | 7 | 10 | 14 | 23 | 33 | 404 | 147 | 2486 | 88 | 180 | 117 | 377 | 3.0 | 0.00087 | 0.676 |
| 3-1 | 975 | 6 | 9 | 13 | 21 | 26 | 196 | 90 | 974 | 50 | 53 | 50 | 65 | 2.9 | 0.00151 | 0.730 |
| 3-5 | 860 | 6 | 10 | 14 | 20 | 23 | 150 | 71 | 860 | 38 | 44 | 39 | 65 | 2.6 | 0.00161 | 0.715 |
| 3-15 | 1180 | 6 | 9 | 13 | 21 | 27 | 203 | 96 | 1178 | 53 | 77 | 61 | 128 | 2.5 | 0.00171 | 0.625 |
| 4-1 | 1318 | 7 | 11 | 14 | 21 | 28 | 232 | 97 | 1316 | 54 | 87 | 65 | 150 | 2.5 | 0.00135 | 0.626 |
| 4-4 | 917 | 5 | 9 | 13 | 18 | 20 | 126 | 56 | 917 | 31 | 42 | 34 | 79 | 2.0 | 0.00179 | 0.591 |
| 4-5 | 956 | 7 | 11 | 15 | 25 | 36 | 188 | 101 | 956 | 67 | 84 | 73 | 113 | 2.8 | 0.00216 | 0.654 |
| 5-1 | 2371 | 7 | 10 | 16 | 23 | 34 | 322 | 128 | 2369 | 68 | 83 | 73 | 116 | 2.8 | 0.00086 | 0.665 |
| 5-4 | 2603 | 7 | 12 | 14 | 21 | 30 | 346 | 143 | 2956 | 80 | 145 | 103 | 267 | 2.7 | 0.00085 | 0.625 |
| 5-5 | 6154 | 7 | 11 | 14 | 24 | 42 | 773 | 259 | 6151 | 165 | 215 | 190 | 262 | 3.4 | 0.00033 | 0.674 |
| All | 31169 | 8 | 15 | 32 | 57 | 81 | 3011 | 583 | 31123 | 346 | 455 | 413 | 523 | 3.6 | 0.00008 | 0.608 |

**^a^** Calculated using the RDP pyrosequencing pipeline including 187 comparable positions after removal of hypervariable positions

**^b^** The MUSCLE alignment includes hypervariable positions

**^c^** Fourty-three sequences corresponding to the reference *E. coli* 16S rDNA positions 300-514 in the MUSCLE alignment were removed from the dataset because they did not fully cover the same reference region using the RDP pyrosequencing pipeline alignment
