# Supplementary material for: Study of inter- and intra-individual variations in the salivary microbiota
Source: BMC Genomics. 2010 Sep 28;11:523. doi: 10.1186/1471-2164-11-523 (PMC2997015; doi:10.1186/1471-2164-11-523)
Supplement: Additional file 3 — Taxonomic positions of universal core and subject-specific phylotypes defined at 100% and 97% identity. [file 1471-2164-11-523-S3.DOC]

| **# of phylotypes defined at 100% identity** | | **# of phylotypes defined at 97% identity** | |
| --- | --- | --- | --- |
| **Universal core [9]** | **Subject(s) specific [69]** | **Universal core [11]** | **Subject(s) specific [17]** |
| Firmicutes [7] | Firmicutes [27] | Firmicutes [7] | Firmicutes [7] |
| *Streptococcus* [3] | *Veillonella* [11] | *Streptococcus* [5] | *Megasphaera* [1] |
| *Veillonella* [3] | *Selenomonas* [3] | *Granulicatella* [1] | *Selenomonas* [1] |
| *Granulicatella* [1] | *Streptococcus* [3] | *Veillonella* [1] | *Streptococcus* [1] |
|  | *Granulicatella* [2] |  | Unclassified Lactobacillales [1] |
|  | *Megasphaera* [2] |  | Unclassified Acidaminococcaceae [1] |
|  | *Anaeroglobus* [1] |  | Unclassified Clostridiaceae [1] |
|  | *Gemella* [1] |  | Unclassified Lachnospiraceae [1] |
|  | Unclassified Lachnospiraceae [2] |  |  |
|  | Unclassified Lactobacillales [2] |  |  |
| Proteobacteria [1] | Proteobacteria [11] | Proteobacteria [2] | Proteobacteria [4] |
| *Campylobacter* [1] | *Haemophilus* [5] | *Campylobacter* [1] | *Heamophilus* [2] |
|  | *Neisseria* [3] | *Heamophilus* [1] | *Neisseria* [2] |
|  | *Lautropia* [2] |  |  |
|  | Unclassified Pasteurellaceae [1] |  |  |
|  | Bacteroidetes [13] |  | Bacteroidetes [2] |
|  | *Prevotella* [7] |  | *Prevotella* [2] |
|  | *Capnocytophaga* [2] |  |  |
|  | *Porphyromonas* [2] |  |  |
|  | *Bergeyella* [1] |  |  |
|  | *Tannerella* [1] |  |  |
|  | Actinobacteria [13] | Actinobacteria [1] | Actinobacteria [2] |
|  | *Actinomyces* [9] | *Actinomyces* [1] | *Atopobium* [1] |
|  | *Atopobium* [2] |  | *Olsenella* [1] |
|  | *Olsenella* [1] |  |  |
|  | Unclassified Actinomycetales [1] |  |  |
|  | Fusobacteria [3] |  | Fusobacteria [1] |
|  | *Fusobacterium* [2] |  | *Selenomonas* [1] |
|  | Unclassified Fusobacteriales [1] |  |  |
| TM7 [1] | TM7 [2] | TM7 [1] | TM7 [1] |

Phylotypes found in the three time-point samples of all subjects are designated as “universal core” phylotypes. Subject specific phylotypes correspond to phylotypes found in the three time-point samples from 1-4 subjects. Number of different phylotypes in genera (italic) and corresponding phyla (underlined) are given in brackets.
