# Supplementary material for: Study of inter- and intra-individual variations in the salivary microbiota
Source: BMC Genomics. 2010 Sep 28;11:523. doi: 10.1186/1471-2164-11-523 (PMC2997015; doi:10.1186/1471-2164-11-523)
Supplement: Additional file 4 — Description of the fasta identifiers in the trimmed dataset. [file 1471-2164-11-523-S4.DOC]

The fasta identifiers of the trimmed dataset (MG-RAST ID:4445506.3) included: 100%-ID phylotype identifier_ordinal number of a given phylotype in a given sample_primer barcode number as described by Hamady et al. [31].

Reverse PCR primers were bar-coded according to samples as follows:

| Primer barcode | Subject | Time-point (days) |
| --- | --- | --- |
| 601 | 1 | 1 |
| 602 | 1 | 5 |
| 603 | 1 | 29 |
| 604 | 2 | 1 |
| 605 | 2 | 7 |
| 606 | 2 | 15 |
| 607 | 3 | 1 |
| 608 | 3 | 5 |
| 609 | 3 | 15 |
| 610 | 4 | 1 |
| 611 | 4 | 4 |
| 612 | 4 | 5 |
| 613 | 5 | 1 |
| 614 | 5 | 4 |
| 615 | 5 | 5 |
